# Encapsulation of Chasteberry (*Vitex agnus castus* L.) Extract by Spray-Drying Followed by Spray-Chilling for Its Application in Dark Chocolate

**DOI:** 10.3390/foods13233742

**Published:** 2024-11-22

**Authors:** Mariana Alejandra Echalar Barrientos, Juliana Peralta, Fabrício Luiz Tulini, Samuel Henrique Gomes de Sá, Marcella Chalella Mazzocato, Marco Antonio Trindade, Valdecir Luccas, Carmen Silvia Favaro-Trindade

**Affiliations:** 1 Faculdade de Zootecnia e Engenharia de Alimentos (FZEA), Universidade de São Paulo (USP), Pirassununga 13635-900, São Paulo, Braziljulianaperalta5493@gmail.com (J.P.); fabricio.tulini@ufob.edu.br (F.L.T.); samuel.sa@usp.br (S.H.G.d.S.); marcella.chalellamazzocato@ucdconnect.ie (M.C.M.); trindadema@usp.br (M.A.T.); 2 Centro das Ciências Biológicas e da Saúde (CCBS), Universidade Federal do Oeste da Bahia (UFOB), Barreiras 47810-047, Bahia, Brazil; 3 School of Agriculture and Food Science, University College Dublin (UCD), Belfield, D04 V1W8 Dublin, Ireland; 4 Cereal Chocotec, Instituto de Tecnologia de Alimentos (ITAL), Campinas 13070-178, São Paulo, Brazil; vluccas@ital.sp.gov.br

**Keywords:** casticin, premenstrual syndrome, vitex, menopause, monk’s pepper, VAC fruits, microencapsulation

## Abstract

Chasteberry extract offers considerable phytotherapeutic benefits, particularly in alleviating premenstrual syndrome (PMS) symptoms. However, its hydroalcoholic nature leads to a bitter taste and a burning sensation, presenting challenges for direct consumption or incorporation into new food products. This study aimed to address these issues by encapsulating concentrated chasteberry extract using spray-drying with Arabic gum, followed by spray-chilling with vegetable fat as carriers. The encapsulated particles were characterized by their morphology, size, and stability, with a specific focus on phenolics and casticin stabilization. The microparticles were incorporated into dark chocolate formulations, and sensory trials conducted with dark chocolate revealed that encapsulation effectively masked undesirable flavors while safeguarding the bioactive compounds. This strategy resulted in a product that demonstrated enhanced stability and sensory appeal. This innovative formulation holds promise for delivering chasteberry phytochemicals that help alleviate PMS symptoms.

## 1. Introduction

*Vitex agnus-castus* L. (VAC) is a bush native to the Mediterranean region and Western Asia, but it is now cultivated globally. Its fruit, widely recognized as chasteberry, vitex, monk’s pepper, or VAC, is used to produce an extract that is a popular herbal remedy for female reproductive conditions in North America and Europe [1].

According to systematic reviews conducted by Van Die et al. [1] and Verkaik et al. [2], studies suggest that the consumption of chasteberry extract may enhance women’s well-being during premenstrual syndrome (PMS) by alleviating symptoms and improving overall quality of life.

Some researchers attribute these health benefits to the phenolic compounds present in chasteberry extract [2,3], while others emphasize casticin [4], a primary phytochemical and chemotaxonomic marker for this genus [5]. Nonetheless, it remains uncertain which specific compounds in chasteberry extract are responsible for its purported positive effects on women’s health.

Chasteberry consumption is primarily associated with its pharmaceutical and health benefits, particularly for disorders related to the female reproductive system [6]. However, the absence of functional products containing chasteberry fruit or its extract prompts the need for new research and the development of alternative ways to consume this product. El-Nawasany [6] developed and evaluated stirred yogurts that contained varying concentrations (0.5%, 1.0%, 1.5%, and 2.0% *w*/*v*) of dried and milled vitex, which mainly included the fruiting tops and leaves.

The production of such products faces challenges due to strong sensory characteristics, such as bitterness and a spicy flavor. One approach to mitigate these sensory issues is the microencapsulation of chasteberry fruit extract. Microencapsulation can help masking unpleasant sensory attributes while protecting the antioxidant capacity of phenolic compounds from degradation caused by unfavorable food processing and storage conditions, such as high temperature, excessive oxygen exposure, light, extreme pH, and high moisture content.

There are several microencapsulation techniques to explore, including spray-drying, spray-chilling, ionic gelation, and complex coacervation. Spray-drying involves nebulizing an emulsion, suspension, or solution in hot air, which promotes rapid dehydration and transforms droplets into microparticles. In contrast, spray-chilling operates similarly but uses cold air instead of hot air. It also employs molten fats as carriers or encapsulants instead of polymers. During the production of spray-chilled microparticles, the mixtures of bioactive material and molten fat are atomized inside a cold chamber, leading to fat crystallization and particle formation. Both methods typically produce microparticles rather than microcapsules, as the active material is dispersed throughout the entire particle volume rather than being surrounded by an encapsulating material. However, by combining both techniques, it is expected that all chasteberry compounds will be retained within double-shell particles, functioning as microcapsules. This would not only protect the compounds but also mask any unwanted sensory effects.

Several studies have indicated an increase in chocolate cravings during the peri-menstrual and pre-menstrual phases [7,8,9,10,11,12]. Michener et al. [13] suggest that these cravings may have a physiological basis. Chocolate serves as a dense source of calories and contains various stimulating substances, including caffeine, theobromine, and sympathomimetic amines such as tyramine and phenylethylamine [14]. Additionally, it features anandamide and two analogs, *N*-oleoylethanolamine and *N*-linoleoylethanolamine, which may contribute to calming and anxiolytic effects [15]

Considering the heightened desire for chocolate during this period and the beneficial properties of chasteberry extract, this study aimed to incorporate both free and microencapsulated chasteberry extract into dark chocolate formulations. To accomplish this, the chasteberry extract was spray-dried, and the resulting microparticles were coated using a spray-chilling technique. The microparticles were characterized based on various parameters, and the chocolates were assessed for bitterness perception and sensory acceptance.

## 2. Material and Methods

### 2.1. Materials

Ripe and dried fruits were obtained from Chá & Cia—Ervas Medicinais (São Paulo, Brazil). Arabic gum (Dinâmica Química Contemporânea Ltda., Indaiatuba, Brazil) and vegetable fat (Triangulo Alimentos, Itápolis, Brazil) with a melting point of ca. 45 °C were used as carriers for the production of microparticles by spray-drying and spray-chilling, respectively. For dark chocolate preparation, sugar (União, São Paulo, Brazil), cocoa liquor (Barry Callebaut, Ilhéus, Brazil), cocoa butter (Barry Callebaut, Ilhéus, Brazil), soy lecithin (Bunge, Brazil), and polyglycerol polyricinoleate (PGPR, Danisco Brasil Ltda., Cotia, Brazil) were used.

### 2.2. Production of Chasteberry Extract and Microparticles

The alcoholic extract of chasteberry was prepared according to Barrientos et al. [16] and analyzed as follows. For the qualitative analysis of phenolic compounds in the vitex fruit extract, the primary fractions were collected using a fraction collector (Shimadzu, Kyoto, Japan) under specific conditions. The mobile phase, with a flow rate of 1 mL/min, consisted of ultrapure water (Direct-Q, Millipore, Rahway, NJ, USA) acidified with formic acid (Sigma-Aldrich, Saint Louis, MO, USA) at pH 3.0 (A) and methanol (Panreac Química SLU, Castellar del Vallès, Spain) (B). The gradient for component B was as follows: 0 min (5%), 0.1–3 min (10%), 3.1–7 min (15%), 7.1–19 min (25%), 19.1–25 min (27%), 25.1–30 min (30%), 30.1–39 min (34%), 39.1–44 min (37%), 44.1–49 min (42%), 49.1–75 min (50%), 75.1–80 min (100%), and at 80.1 min returning to (5%). The column oven was maintained at 35 °C, and 30 µL of the sample was injected [17].

Next, the extract was concentrated to achieve 20 g of solids per 100 g of extract using a rotary evaporator at 45 °C. Next, the concentrated extract was mixed with Arabic gum at a concentration of 5 g per 100 g of extract. This feed material was then atomized using spray-dryer equipment (Model MSD 1.0, Labmaq, Ribeirão Preto, Brazil) under the operational conditions outlined by Barrientos et al. [16]. After spray-drying, 5 g, 10 g, and 15 g of powders were blended with 50 g of vegetable fat that had been melted at 60 °C, resulting in three formulations. These blends were atomized using the same operational conditions as the initial spray-drying process, except for the inlet air temperature, which was set to 14 °C. For the control formulation, the concentrated extract without Arabic gum was also spray-dried under the same conditions. This material was referred to as the free extract (FC), and it was used for dark chocolate production.

### 2.3. Characterization of Powders and Their Particles

The powders obtained from the two techniques were stored at 25 °C with 38% relative humidity (RH), in the presence of oxygen but without exposure to light, for up to 120 days. Moisture, water activity (Aw), particle size, and X-ray diffraction samples were analyzed at day zero (i.e., on the same day the extract was encapsulated) and after the 120-day storage period.

#### 2.3.1. Moisture and Water Activity (Aw)

Moisture content was determined using a moisture analyzer (Ohaus, model MB 35, Canton, OH, USA). Water activity (Aw) was measured using Aqualab equipment (Decagon Devices, Pullman, WA, USA).

#### 2.3.2. Particle Size

Particle sizes were analyzed according to Salvim et al. [18] using laser diffraction (Sald-201V, Shimadzu, Kyoto, Japan). Absolute ethanol served as the dispersant for the particles, and particle sizes were expressed as the De Brouckere mean diameter (D4,3).

#### 2.3.3. X-Ray Diffraction (XRD)

The polymorphic forms of the powders were evaluated using XRD analysis with an AXS Analytical X-Ray Systems Siemens (Munich, Germany). The powders were scanned from 5° to 55° of 2θ at a rate of 3°/min, as described by Xiao et al. [19].

#### 2.3.4. Scanning Electronic Microscopy (SEM)

Particle morphology was assessed using a Tabletop Scanning Electron Microscope (SEM) from Hitachi (Tokyo, Japan) at magnifications of 500× or 100× without gold-coating the particles.

#### 2.3.5. Thermogravimetric Analysis of Powders

This analysis was performed with Shimadzu TGA-50 equipment (Japan), calibrated at a heating rate of 10 °C/min using high-purity calcium oxalate monohydrate.

#### 2.3.6. Total Phenolic Compounds of Powders

The Folin–Ciocalteu reagent was utilized to quantify phenolic compounds in the powders. The results were expressed in milligrams of gallic acid equivalents (GAE) per gram of dry matter, following the method outlined by Singleton et al. [20], with some modifications. The reaction mixture was prepared using 0.25 mL of the sample, 2 mL of distilled water, and 0.25 mL of the Folin–Ciocalteu reagent. After incubating the mixture at room temperature for 3 min, 0.25 mL of a saturated sodium carbonate (Na_2_CO_3_) aqueous solution was added. This was followed by further incubation at 37 °C in a water bath for 30 min. The absorbance was measured at 750 nm using an Ultrospec 2000 spectrophotometer (Amersham Pharmacia Biotech INC, Piscataway, NJ, USA), and a standard curve of gallic acid ranging from 0.890909 to 4.454545 µg/mL was used to determine the total phenolic concentration. Results were expressed as mg GAE/g of sample on a dry basis.

#### 2.3.7. Casticin Quantification in the Powders by HPLC

Casticin quantification was conducted based on the method described by Hoberg et al. [5] using a Shimadzu high-performance liquid chromatography (HPLC) system, model Prominence, equipped with a diode array detector. The column oven was kept at 30 °C, and 10 µL of the sample diluted in methanol was injected into the equipment. The separations were executed at a flow rate of 1 mL/min, with the mobile phase consisting of methanol (A) and a 0.5% phosphoric acid solution (B), applied in a gradient as follows: from 0–13 min (50:50), at 13 min (65:35), from 13.1–18 min (100:0), and from 18–23 min (50:50). Chromatograms were acquired at a wavelength of 256 nm and the results were expressed as µg of casticin per gram of sample on a dry basis.

#### 2.3.8. Evaluation of the Stability of Total Phenolic Compounds and Casticin

The encapsulated extract of chasteberry was portioned into glass vials with plastic lids. These vials were stored in a controlled environment at 25 °C and 38% relative humidity (RH), with exposure to oxygen but protected from light for up to 120 days. The total phenolic and casticin contents were evaluated at day zero and after 15, 30, 60, 90, and 120 days of storage, according to the methods outlined in Section 2.3.6 and Section 2.3.7, respectively.

### 2.4. Production of Dark Chocolate Added of Chasteberry Extract Free and Encapsulated

The production of dark chocolate bars incorporating free and encapsulated chasteberry extract was carried out using a conventional manufacturing system. This process took place at the Ceral Chocotec pilot plant of the Instituto de Tecnologia de Alimentos in Campinas, Brazil. The formulation used consisted of the following ingredients: 47% sugar (*w*/*w*), 40% cocoa liquor (*w*/*w*), 10% cocoa butter (*w*/*w*), 0.3% soy lecithin (*w*/*w*), 0.2% PGPR (*w*/*w*), and 2.5% (*w*/*w*) of either free chasteberry extract (along with Arabic gum and vegetable fat in the same proportions as found in the encapsulated extract) or encapsulated chasteberry extract. Before refining, the ingredients were mixed in an agitated jacketed tank (Inco, Rodenbach, Germany) with water circulation maintained at 45 °C. After the lipid phase melted completely and the mixture was homogenized, it was processed in a five-roller refiner (JAF Inox, Duyvis Wiener, Tambaú, Brazil) and cooled by a chiller (MeCalor, São Paulo, Brazil) at approximately 7 °C. The pressure between the cylinders was adjusted to achieve maximum particle sizes smaller than 28 µm, which were measured using a digital micrometer. The blend was then conched at 65 °C in a homogenizing conching machine (JAF Inox, Duyvis Wiener, Tambaú, Brazil), which performed three stages: dry conching (4 h at 60 Hz), plastic conching (19.5 h at 60 Hz), and liquid conching with emulsifier addition (30 min at 30 Hz). Following this, the chocolate was tempered or pre-crystallized in a benchtop tempering machine (ACMC, New York, NY, USA), starting at 40 °C and cooling down to 28 °C at a rate of 2 °C/min. This process is ideal for obtaining crystals in the Beta V polymorph.

The free and microencapsulated chasteberry extracts were added during the tempering stage when the chocolate temperature reached 35 °C, ensuring the integrity of the microparticles. The pre-crystallized chocolate was then poured into polycarbonate molds, and air bubbles were removed through vibration. Finally, the chocolate bars were crystallized in a cooling tunnel (SIAHT) operating at temperature ranges of 15–17 °C for the inlet and outlet and 11–13 °C in the middle of the tunnel. These temperature conditions allowed for the consolidation of the lipid matrix in the Beta Form, providing the chocolate with the desired physical properties, such as hardness and melting characteristics. After the cooling process, the products were unmolded, wrapped in aluminum foil, and stored in a BOD oven (ELETROlab^®^, São Paulo, Brazil) at a controlled temperature of 19 ± 1 °C while being protected from light and humidity. This storage condition is crucial for the complete formation of the desired crystal lattice in the chocolate.

It is important to note that three treatments were prepared: one containing the encapsulated powder (15 g of spray-dried extract per 50 g of vegetable fat), a control with no addition of extract or vegetable fat, and the final treatment consisting of the free extract combined with the carrier agents (Arabic gum and vegetable fat) used to create the encapsulated extract, mixed in the same proportions as the particles but mechanically blended into a powder. Additionally, a previous study obtained the free extract through a spray-drying process without any carrier additives [16]. The chocolate containing the encapsulated and free chasteberry extracts was designated “EC” and “FC”, respectively.

#### 2.4.1. Stability of Casticin and Total Phenolic in Chocolates

The stability of phenolic compounds and casticin in chocolates wrapped in aluminum foil and stored at 22 °C was monitored over a period of 0, 7, 15, 30, 45, and 60 days of storage. The quantification methods used were previously described in Section 2.3.6 and Section 2.3.7. To extract the phenolic compounds and casticin, the chocolate samples were first degreased and then extracted using an 80% (*v*/*v*) ethanol solution, following the protocols established by Adamson et al. [21] and Alanón et al. [22]. Specifically, 3 g of ground chocolate was mixed with 10 mL of n-hexane. This mixture was homogenized using a vortex, placed in an ultrasonic bath for 5 min, and then centrifuged at 2935× *g* for 5 min. The procedure was repeated, adding only 5 mL of n-hexane for the second extraction. The samples were then dried to remove any residual n-hexane. To maximize the recovery of total phenolic compounds and casticin, extraction was performed twice. After defatting the chocolate, 2.5 mL of the 80% (*v*/*v*) alcoholic solution was added to the sample. This mixture was again homogenized by vortexing and subjected to ultrasonic treatment for 10 min, followed by centrifugation at 2935× *g* for 5 min, resulting in a total of 5 mL of final extract. For the quantification of casticin, the extract was filtered. In contrast, the measurement of phenolic compounds involved diluting the chasteberry extract in a ratio of 0.8:10 using the 80% (*v*/*v*) ethanol solution.

#### 2.4.2. Sensory Evaluation

Two sensory evaluation tests were carried out: (1) paired comparison test and (2) acceptance test using a 9-point hedonic scale. The sensory evaluation study was approved by our institution’s research ethics committee, receiving the protocol number (CAAE 56690216.2.0000.5422). Additionally, all panelists provided their consent by signing a consent form. All samples were served in disposable 50 mL plastic cups coded with three random digits, accompanied by a biscuit and a glass of water, allowing panelists to cleanse their palates between each tasting.

Two paired comparison tests (two-tailed) were conducted, following the methodology of Meilgaard et al. [23], to evaluate the effectiveness of the microencapsulation process in masking or reducing the bitterness and burning sensation associated with chasteberry extract. For both, the same group of participants took part, who were selected based on their sensitivity to spicy (15 participants) and bitterness (17 participants) sensations.

In the first paired comparison test, panelists compared encapsulated and non-encapsulated powders, as follows: (i) the encapsulated chasteberry extract, and (ii) the sample featuring free freeze-dried chasteberry extract combined with the encapsulating agents in the same proportions as found in the encapsulated particles, but mechanically mixed into a powder.

In the second test, panelists evaluated the two chocolates (dark chocolate bars incorporating free and encapsulated chasteberry extract) produced as described in Section 2.4.

Furthermore, sensory acceptance of the chocolates was assessed, according to Meilgaard et al. [22], with a group of 122 untrained women aged between 18 and 60 years previously recruited among regular consumers of chocolate. Besides the chocolate bars with free and encapsulated chasteberry extract, consumers also evaluated a commercial chocolate sample (Control sample). All samples (control, EC, and FC) were presented in the same conditions as in the previous test. The participants evaluated product acceptance using a 9-point hedonic scale (1—“Dislike extremely” to 9—“Like extremely”) for the attributes of flavor, texture, color, aroma, and overall acceptability. To determine the product’s purchase intention, a 5-point scale was utilized, where 1 indicated “Definitely do not buy” and 5 indicated “Definitely buy.” The acceptance index (AI) was calculated by taking the average score of all evaluated attributes, dividing it by the maximum score of the hedonic scale (9), and multiplying the result by 100 [24].

### 2.5. Statistical Analyses

Statistical analyses were performed using one-way ANOVA followed by Tukey’s test, considering significant differences when *p* < 0.05 (SAS software, version 9.2).

## 3. Results and Discussion

### 3.1. Characterization of Extract, Powders and Their Particles

**Figure 1:**
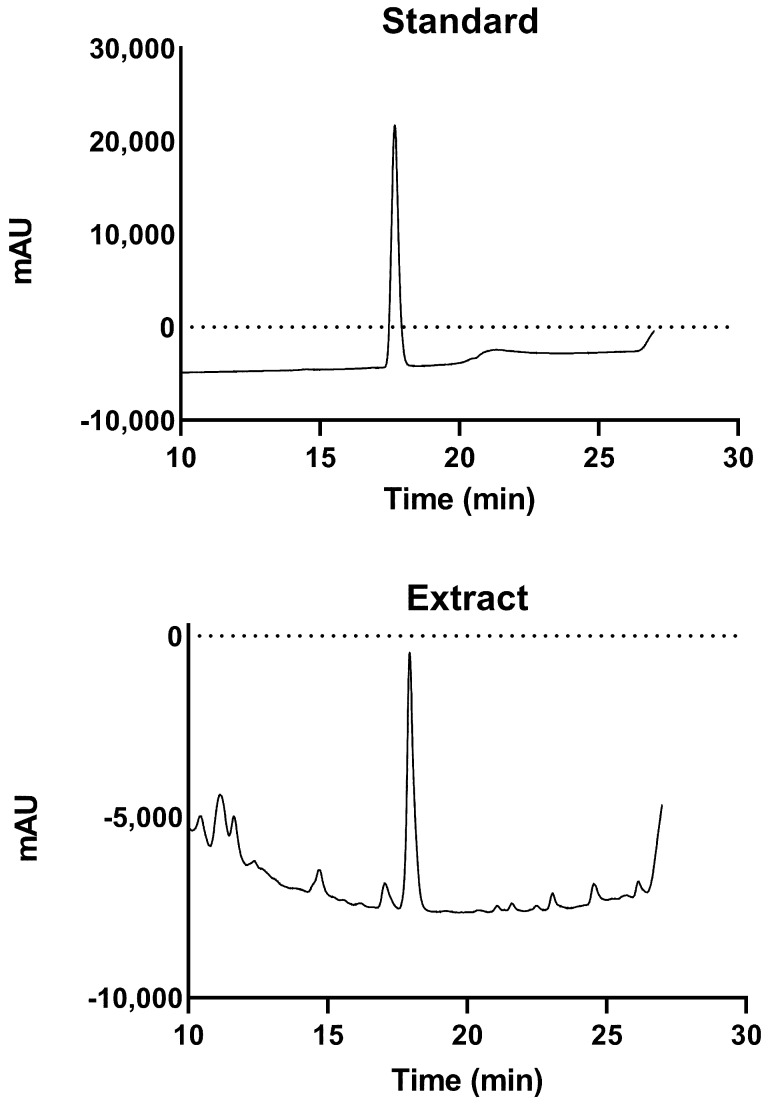
Chromatographic analysis of chasteberry extract, showing the casticin peak at 17 min. Upper chromatogram was made using an external standard for casticin.

Figure 1 illustrates the chromatogram obtained through high-performance liquid chromatography, following the methodology established by Fukahori et al. [17] for analyzing phenolic compounds. The peaks at approximately 17 min, when compared to the external standard chromatogram, were identified as casticin, similar to the results reported by Fukahori et al. [17].

Arabic gum was selected as a carrier in the spray-drying process due to its affordability, scalability, and its designation as Generally Recognized as Safe (GRAS). It plays a vital role in protecting active compounds from degradation while enhancing the solubility and dispersion of the encapsulated materials. Arabic gum also stabilizes the particles throughout the process, preventing clumping and preserving the properties of the bioactive compounds. Subsequently, the particles obtained through the encapsulation of vitex extract via spray-drying were subjected to the spray-chilling process.

**Figure 2:**
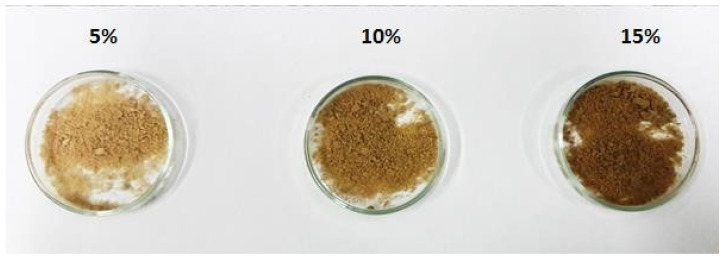
The appearance of powders produced by spray-drying and coated by spray-chilling, using 5, 10, and 15 g of spray-dried chasteberry extract per 50 g of vegetable fat.

The particles produced in this study yielded darker powders, correlating with the higher concentrations of chasteberry extract used in the formulation (Figure 2). This phenomenon can be attributed to the presence of numerous flavonoids in the chasteberry extract, which are pigments that impart a light brown color. Consequently, a higher flavonoid content in the formulation results in powders with more intense hues. Similar findings were reported by Mazzocato et al. [25], who examined particles produced by spray-chilling that were loaded with vitamin B12. Through color analysis, they found that formulations with elevated vitamin B12 concentrations displayed “L”, “a”, and “b” parameters indicative of more intense staining and a reddish hue in samples with greater vitamin content.

#### 3.1.1. Moisture and Water Activity (Aw)

Aw and moisture are critical parameters to assess during the storage of powders. A comparison of the moisture values on the first day versus after 120 days of storage revealed a significant increase for all formulations, which is expected given that the samples were stored for 120 days at 38% relative humidity (RH). In contrast, the water activity (Aw) remained stable only in the formulation containing 15% chasteberry extract. Meanwhile, the formulations with 5% and 10% chasteberry extract exhibited a decrease and an increase in Aw, respectively, after 120 days. However, from a technological standpoint, the variations in moisture and water activity can be considered negligible; moisture levels below 20% are regarded as very low, and Aw values below 0.6 ensure microbiological stability for the powders. The minor fluctuations in these parameters can be attributed to the composition of the particles, including the presence of hydrophobic vegetable fat that is unable to bind water.

#### 3.1.2. Particle Size

**Figure 3:**
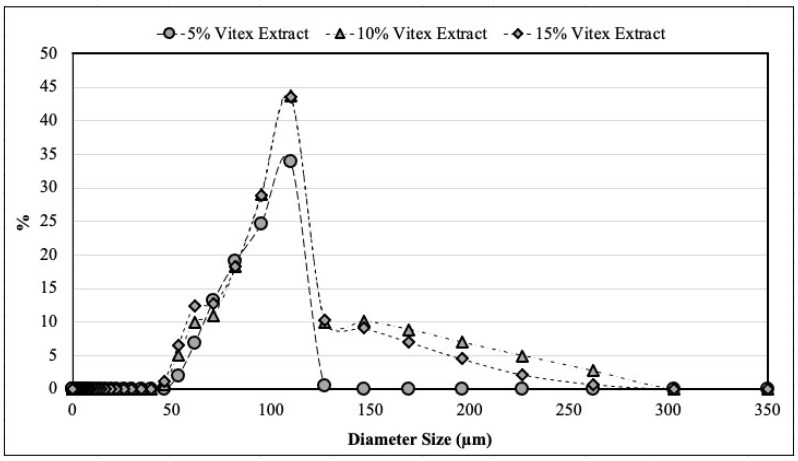
Size distribution of microparticles produced by spray-drying and coated by spray-chilling, using 5, 10, and 15 g of spray-dried chasteberry extract per 50 g of vegetable fat.

Particle size can significantly impact the sensory characteristics of foods, particularly in relation to texture. Therefore, it is essential to assess this parameter in the powders used in this product. Additionally, the average particle size may vary depending on factors such as temperature, air velocity, lipid composition, filling composition, and storage conditions. Figure 3 illustrates the particle size distribution on the first day of storage. In the beginning, most particles across all formulations had a diameter of 109.822 µm. However, after 120 days of storage, powders made with 5% chasteberry extract exhibited particle diameters of approximately 261.95 µm, while the other formulations had diameters of around 302.79 µm. Regarding the D4,3 values on the first day of storage, particles produced with 5%, 10%, and 15% chasteberry extract presented values of 86.6 µm, 78.4 µm, and 88.1 µm, respectively. After 120 days, the particles with 5%, 10%, and 15% chasteberry extract showed D4,3 values of 254.5 µm, 268.9 µm, and 289.2 µm, respectively. These findings indicate that the particles agglomerated during storage, and this aggregation can be attributed to fat melting and recrystallization.

Tulini et al. [26] reported similar results for particles containing spray-dried cinnamon extracts, which displayed an unimodal size distribution between 60 and 130 µm, along with an increase in D4,3 values after 90 days of storage. Microencapsulation via spray-chilling typically produces particles with an average diameter ranging from 20 to 200 µm. This outcome may relate to the viscosity of the solutions before atomization. According to Albertini et al. [27] and Zuidam & Shimoni [28], highly viscous solutions yield particles between 150 and 250 µm, while low-viscosity solutions result in particles between 75 and 150 µm.

#### 3.1.3. XRD

**Figure 4:**
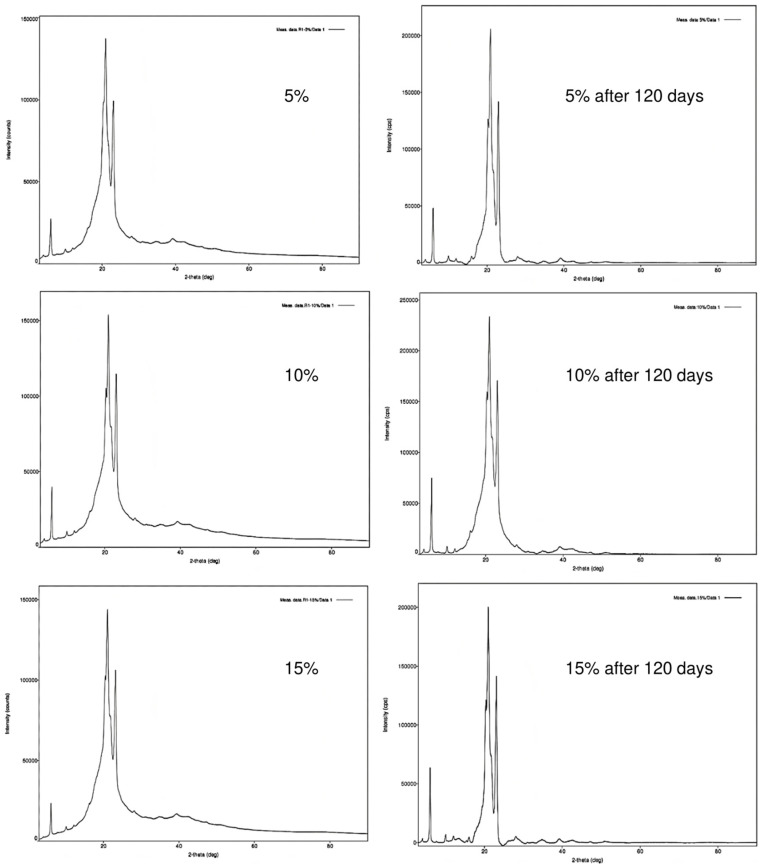
X-ray diffractograms of powders produced by spray-drying and coated by spray-chilling with 5, 10, and 15 g of spray-dried chasteberry extract per 50 g of vegetable fat.

Another critical issue concerning lipid particles is that triacylglycerols exhibit different polymorphic forms; these are solid phases with similar chemical compositions but differing crystal structures due to their complex molecular nature [29]. Lipids can crystallize in three polymorphic forms: alpha (the least stable), beta-prime, and beta (the most stable). Consequently, the sensory properties of foods can be influenced by the polymorphic phase of the lipid matrix [30]. In our study, all particles exhibited a consistent pattern of peaks that remained stable over 120 days (Figure 4). There was only a slight deviation and reduction in intensity, typical of long storage periods, but this did not alter the overall pattern of crystallization. This stability indicates that the particles retained the same crystalline forms over time, preventing the expulsion of extract compounds that could occur during phase transitions of polymorphic lipids. Additionally, as shown in the diffractograms presented in Figure 4, the particles are composed of β′ type crystals, which aligns with expectations since they were produced using vegetable fat. According to DeMan [31], this type of crystal is typical of vegetable fats or “shortenings”. This finding supports the work of Tulini et al. [32], who utilized the same vegetable fat to co-encapsulate spray-dried cinnamon extract and tocopherol through spray-chilling.

Similarly, Gamboa et al. [29] produced particles with tocopherol using the spray-chilling method and noted that the prominent peaks corresponded to angles between 22° and 23° of 2θ, which relate to the most stable form of fat crystals (beta type). Furthermore, it is important to highlight that the stability of fat crystals minimizes the likelihood of fat bloom in the produced chocolate, especially given that the samples are stored under favorable and controlled temperature conditions.

#### 3.1.4. SEM

**Figure 5:**
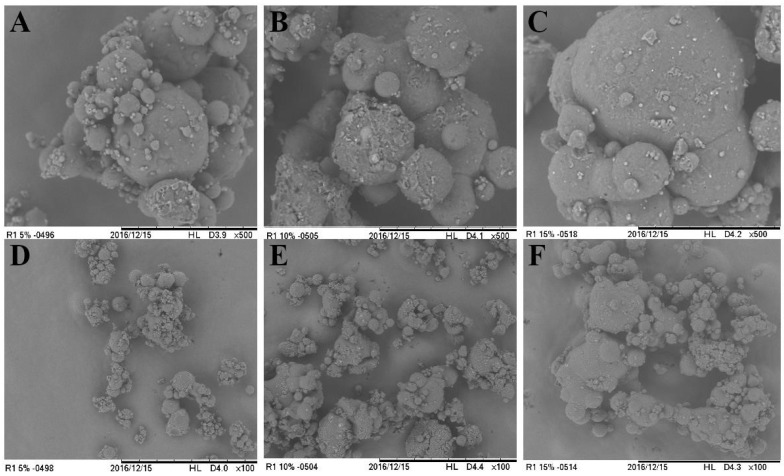
Scanning electronic micrographs (500× and 100× magnification) of particles produced by spray-drying and coated by spray-chilling; (**A**) particles produced with 5 g of spray-dried chasteberry extract per 50 g of vegetable fat and 500x magnification; (**B**) particles produced with 10 g of spray-dried chasteberry extract per 50 g of vegetable fat and 500× magnification; (**C**) particles produced with 15 g of spray-dried chasteberry extract per 50 g of vegetable fat and 500× magnification; (**D**) particles produced with 5 g of spray-dried chasteberry extract per 50 g of vegetable fat 100× magnification; (**E**) particles produced with 10 g of spray-dried chasteberry extract per 50 g of vegetable fat 100× magnification; (**F**) particles produced with 15 g of spray-dried chasteberry extract per 50 g of vegetable fat—100× magnification.

SEM was applied to evaluate the external morphology and macrostructure of the particles. As shown in Figure 5, the particles produced exhibited a spherical shape and a smooth, continuous surface. This finding aligns with observations reported in similar studies [26,30,33]. Furthermore, the continuous surfaces can be attributed to the spray-chilling atomization process, which does not involve solvent evaporation—unlike spray-drying, for example [34]. This process helps maintain the integrity and continuous surface of the microspheres.

#### 3.1.5. Thermogravimetric Analysis of Powders

**Figure 6:**
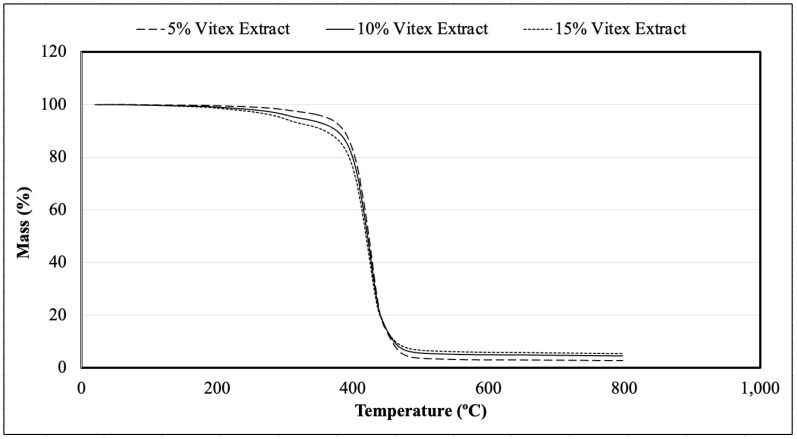
Mass variation according to the temperature applied in samples produced with 5, 10, and 15 g of spray-dried chasteberry extract per 50 g of vegetable fat.

The analysis of thermogravimetric data is essential for assessing the mass variation of a material over time or temperature. As shown in Figure 6, all formulations displayed similar thermal behavior. Initially, the mass composition was 100%, but, at approximately 400 °C, degradation of fats and chasteberry extract was observed, indicating a link between high temperatures and thermal decomposition. Sillick and Gregson [35] studied the encapsulation of aromas using erythritol anhydrides through spray-chilling, noting that degradation of the samples occurred at an average temperature of 125 °C. Bianchi et al. [36] reported that mass losses below 100 °C were primarily related to water loss. In the range of from 200 to 350 °C, the losses were associated with the volatilization of volatile components, while declines between 300 and 500 °C were linked to the degradation and decomposition of various compounds.

### 3.2. Stability of Total Phenolics and Casticin in the Powders

The method that employs the Folin–Ciocalteu reagent, as detailed in Section 2.3.6, resulted in the establishment of a linear equation defined by y = −0.0405 + 172.1837x. This equation demonstrates a strong correlation, as indicated by an R^2^ value of 0.9974, suggesting a highly reliable relationship between the variables under study. According to the data presented in Table 1, formulations containing 5% and 10% chasteberry extract demonstrated a decrease in phenolic content after 15 days of storage, particularly the formulation with 5% extract. However, both formulations exhibited no changes in phenolic levels between days 15 and 120. In contrast, the formulations with 15% chasteberry extract showed high stability of phenolic compounds throughout the entire 120 days of storage. Similarly, Table 2 indicates that the casticin content in the formulation with 15% chasteberry extract remained constant for up to 120 days, while the formulations with 5% and 10% chasteberry extract experienced some fluctuations during the 120-day storage period. Barrientos et al. [16] reported that phenolic compounds maintained high stability in microparticles loaded with chasteberry extract powders produced by spray-drying at temperatures of 130 °C and 160 °C, using Arabic gum as the carrier, which supports the findings of this study. However, it is important to note that spray-dried microparticles are highly water-soluble and unable to mask unpleasant flavors. This limitation has led to the exploration of alternative technologies, such as combining spray-drying with spray-chilling, to produce double-shell microparticles.

### 3.3. Stability of Total Phenolics and Casticin in the Chocolates

During the storage of chocolates at 22 °C, the degradation of phenolic compounds occurred across all formulations over a 60-day period, particularly after the first 30 days. This degradation resulted in a reduction of phenolic content to less than half of its initial level (see Table 3). Notably, there was no significant difference in phenolic content among the various chocolate formulations, suggesting that the addition of both free and encapsulated chasteberry did not enhance the original phenolic content in the chocolates. This finding may be attributed to the low concentration of extract added. In summary, the levels of phenolic compounds in all dark chocolate formulations exhibited low stability over the 60-day period, and the incorporation of free or encapsulated chasteberry extract did not have an impact on this stability.

Regarding the stability of casticin (Table 4), a significant decline in casticin levels was noted in EC chocolate after 15 days, whereas no changes were observed in the FC chocolate during the entire 60-day period. It is important to mention that the initial casticin levels in FC chocolates were lower than those in EC chocolates, likely due to the loss of casticin during the incorporation of the free extract into the chocolate. Furthermore, casticin demonstrated greater stability over the storage period compared to the total phenolic content. By the 60th, phenolic compounds had decreased to less than half in all formulations, while the casticin content in EC chocolates showed only a 25% reduction.

### 3.4. Sensory Evaluation

The formulation of particles containing 15% chasteberry extract was selected to assess the encapsulation potential for masking or mitigating the burning sensation and bitterness associated with chasteberry extract. This formulation demonstrated superior storage stability and effective retention of total phenolic compounds, including casticin. Results indicated that panelists were able to detect significant differences between the microencapsulated extract and the mechanically mixed powder in terms of bitterness and spiciness (Table 5) through the paired comparison tests. This suggests that the chasteberry extract-loaded particles were effective in masking or reducing both the burning sensation and bitterness. However, when conducting the same sensory test (paired comparison) with the chocolates containing free form (FC) and encapsulated chasteberry extract (EC), no significant differences were detected by panelists for both attributes (bitterness and spiciness). Despite not being significantly different, the EC chocolate was found to be less bitter and spicy compared to the FC chocolate for most of the panelists, indicating that the techniques employed to produce the microparticles successfully masked the distinctive taste of chasteberry.

A sensory acceptance test was conducted with 122 female participants. Among them, 88% were aged between 18 and 25 years, with 44% preferring milk chocolate and 42% preferring dark chocolate. Additionally, 60% of them consumed chocolate on a weekly basis. Participants were asked if they engaged in activities to alleviate PMS symptoms and whether they would consider consuming functional food products designed to address these issues. The responses revealed that 49% of participants did not engage in any specific activities, 33% exercised regularly, and 16% took medications. Notably, 97% indicated a willingness to consume a functional product aimed at alleviating PMS symptoms, highlighting the potential for successfully launching a product with these attributes.

The results presented in Table 6 indicate that all grades were 6 or above (“I liked it a little”), signifying positive acceptance of the chocolates by participants. Furthermore, all attributes displayed a significant difference between the free chasteberry (FC) and encapsulated chasteberry (EC) chocolates compared to the control chocolate, which received the highest score, suggesting a willingness to purchase it. While there were no significant differences between FC and EC chocolates in terms of flavor, aroma, and color, they did differ statistically in texture. However, the overall acceptance did not vary significantly, allowing us to conclude that chocolates infused with chasteberry (whether in the free or encapsulated form) had slightly lower acceptance than the standard chocolate but still enjoyed high approval.

Interestingly, FC chocolates were better received in terms of texture, suggesting that the encapsulated particles may have negatively impacted this attribute. Additionally, the intention to purchase varied across the samples, with the control chocolate receiving the highest score, followed by the FC and then the EC chocolates. This variation, in general acceptability, seems to have been influenced by the texture.

The acceptance index (AI) ranged from 78% to 88%. According to Dutcosky [24], products are deemed well-accepted if their AI exceeds 70%. Therefore, despite the slightly inferior performance of the chocolates containing chasteberry extract, the AI results indicate that all chocolates produced were well-accepted. Consequently, the products developed in this study, once they undergo clinical trials, could serve as promising alternatives in the market aimed at alleviating premenstrual symptoms.

Considering just the results of sensory acceptance, the use of chasteberry extract encapsulated in dark chocolate formulations seems dispensable; however, the technique of encapsulation used was effective in protecting casticin and in masking the extract’s bitterness and burning sensation. In addition, although the cost has not been evaluated, the techniques of spray-drying followed by spray-chilling that were applied in this study for encapsulation of the extract are fast, highly productive, and continuous processes, and they do not require expensive carriers; so, the encapsulated extract is probably slightly more expensive than the free one studied (produced by freeze drying), but it still is worthwhile.

For future studies, it could be interesting to evaluate the effectiveness of using particles produced just by spray-drying in minimizing undesirable sensory aspects and in protecting the bioactive compounds of the extract because these particles certainly would be less expensive than the ones produced using the combination of spray-drying and spray-chilling techniques and also cheaper than the ones produced by freeze-drying.

## 4. Conclusions

Microencapsulation techniques applied to chasteberry extract proved to be efficient for protecting casticin, one of the compounds related to relieving PMS symptoms, but it was inefficient in protecting the total phenolic content. Additionally, while microencapsulation successfully reduced the burning sensation and bitterness of the extract, chocolates made with this encapsulated material received lower acceptance for their texture compared to those made with the free-form extract. To enhance the texture of these chocolates, incorporating solid aggregates, such as nuts, crispy rice, or dried fruits, after the tempering process could be a viable solution. Interestingly, the overall acceptance and purchase intent for chocolates made with free-form chasteberry were similar to those made with microparticles of the extract.

Therefore, it is advisable to produce functional chocolates using only the extract obtained through spray-drying, eliminating the encapsulation step. This approach would not only be more cost-effective and quicker but would still provide the phytotherapeutic benefits of casticin for women experiencing premenstrual syndrome or menopause. In summary, prioritizing the use of chasteberry extract obtained by spray-drying would lead to a more efficient production process while maintaining its health benefits.

## Figures and Tables

**Table 1:** Content of total phenolics (mg GAE/g) in particles produced by spray-chilling and loaded with spray-dried chasteberry extract at concentrations of 5, 10, and 15% for up to 120 days of storage.

Days	5%	10%	15%
0	5.7 ± 0.7 ^Ba^	5.6 ± 1.1 ^Aa^	6.5 ± 0.4 ^Aa^
15	2.6 ± 0.1 ^Cbc^	4.5 ± 0.4 ^Bb^	6.0 ± 0.3 ^Aab^
30	2.6 ± 0.2 ^Cbc^	4.6 ± 0.1 ^Bb^	5.7 ± 0.7 ^Aab^
60	2.5 ± 0.4 ^Cbc^	3.4 ± 0.5 ^Bc^	5.2 ± 0.4 ^Ab^
90	2.1 ± 0.5 ^Cc^	4.2 ± 0.4 ^Bbc^	5.3 ± 1.0 ^Ab^
120	2.7 ± 0.6 ^Cbc^	4.4 ± 0.6 ^Bbc^	6.0 ± 0.2 ^Aab^

Mean ± standard deviation (n = 6 replicates). Different uppercase letters in the same row and lowercase letters in the same column indicate a significant difference between the samples (*p* < 0.05).

**Table 2:** Content of casticin (µg casticin/g of sample) in particles produced by spray-chilling and loaded with spray-dried chasteberry extract at concentrations of 5, 10, and 15% for up to 120 days of storage.

Days	5%	10%	15%
0	21.0 ± 6.0 ^Ba^	32.5 ± 14.5 ^Ba^	47.0 ± 5.1 ^Aa^
15	19.5 ± 2.5 ^Bab^	34.7 ± 9.4 ^Ba^	45.6 ± 11.7 ^Aa^
30	14.9 ± 2.4 ^Cab^	30.4 ± 3.7 ^Ba^	51.9 ± 3.0 ^Aa^
60	13.4 ±3.1 ^Bbc^	33.0 ± 5.1 ^Aa^	41.1 ± 12.5 ^Aa^
90	11.9 ± 2.9 ^Cc^	29.6 ± 2.8 ^Ba^	44.7 ± 5.9 ^Aa^
120	14.9 ± 3.3 ^Bbc^	32.5 ± 3.6 ^Aa^	39.1 ± 15.6 ^Aa^

Mean ± standard deviation (n = 3 replicates). Different uppercase letters in the same row and lowercase letters in the same column indicate a significant difference between the samples (*p* < 0.05).

**Table 3:** Content of total phenolics (mg GAE/g) in chocolates with free (FC) and encapsulated chasteberry extract (EC) for up to 60 days of storage, compared to the control chocolate.

Days	Control	EC	FC
0	7.8 ± 0.1 ^Aa^	7.8 ± 1.2 ^Aab^	7.4 ± 0.5 ^Ab^
7	7.0 ± 0.6 ^Aa^	6.5 ± 1.0 ^Ab^	6.0 ± 0.8 ^Ac^
15	8.0 ± 0.4 ^Aa^	8.3 ± 0.6 ^Aa^	8.5 ± 1.0 ^Aa^
30	4.3 ± 0.1 ^Ab^	3.0 ± 1.0 ^Bcd^	4.6 ± 0.3 ^Ad^
45	3.5 ± 1.8 ^Abc^	4.4 ± 0.4 ^Ac^	4.6 ± 0.07 ^Ad^
60	2.6 ± 1.1 ^Ac^	2.2 ± 0.7 ^Ad^	2.2 ± 0.3 ^Ae^

Mean ± standard deviation (n = 6 replicates). Different uppercase letters in the same row and lowercase letters in the same column indicate a significant difference between the samples (*p* < 0.05).

**Table 4:** Content of casticin (µg casticin/g) in chocolates with free (FC) and encapsulated chasteberry extract (EC) for up to 60 days of storage.

Days	EC	FC
0	7.6 ±0.6 ^Aa^	5.6 ± 0.2 ^Ba^
7	6.9 ± 0.3 ^Aab^	5.0 ± 0.2 ^Ba^
15	5.5 ± 0.4 ^Ac^	4.9 ± 0.2 ^Aa^
30	6.1 ± 0.6 ^Abc^	4.6 ± 0.5 ^Ba^
45	5.4 ± 0.4 ^Ac^	5.3 ± 0.8 ^Aa^
60	5.7 ± 0.5 ^Abc^	5.1 ± 0.4 ^Aa^

Mean ± standard deviation (n = 3 replicates). Different uppercase letters in the same row and lowercase letters in the same column indicate a significant difference between the samples (*p* < 0.05).

**Table 5:** Results for sensorial analyses of chocolates with free (FC) and encapsulated chasteberry extract (EC), as well as powders produced the encapsulated extract (EC) and with the spray-dried extract, vegetable fat, and Arabic gum (exactly in the same proportion as these appear in the formulation, but mechanically mixed—FC).

	Powder		Chocolate	
	Bitterness	Spicy	Bitterness	Spicy
FC	17 ^A^	13 ^A^	10	11
EC	0 ^B^	2 ^B^	7	4

Different letters in the column indicate a significant difference between the samples (*p* < 0.05).

**Table 6:** Average grades assigned by chocolate consumers in the acceptance test and purchase intention for the different formulations of chocolate (commercial sample (control), with free (FC) and encapsulated chasteberry extract (EC)).

Chocolate Sample	Flavour	Aroma	Texture	Colour	Overall Acceptability	Overall Average	Acceptance Index (%)	Purchase Intention
Control	8 ± 1 ^A^	8 ± 1 ^A^	8 ± 1 ^A^	8 ± 1 ^A^	8 ± 1 ^A^	8	88.9%	5 ± 1 ^A^
EC	7 ± 2 ^B^	7 ± 1 ^B^	6 ± 1 ^C^	8 ± 1 ^B^	7 ± 2 ^B^	7	78%	3 ± 1 ^C^
FC	7 ± 2 ^B^	7 ± 1 ^B^	7 ± 2 ^B^	8 ± 1 ^B^	7 ± 2 ^B^	7.2	80%	4 ± 1 ^B^

Mean ± standard deviation (n = 122 replicates). Different letters in the column indicate a significant difference between the samples (*p* < 0.05).

## Data Availability

The original contributions presented in the study are included in the article, further inquiries can be directed to the corresponding author.

## References

[1] van Die M., Burger H., Teede H., Bone K. (2012). Vitex Agnus-Castus Extracts for Female Reproductive Disorders: A Systematic Review of Clinical Trials. Planta Med..

[2] Verkaik S., Kamperman A.M., van Westrhenen R., Schulte P.F.J. (2017). The Treatment of Premenstrual Syndrome with Preparations of Vitex Agnus Castus: A Systematic Review and Meta-Analysis. Am. J. Obstet. Gynecol..

[3] Li S., Qiu S., Yao P., Sun H., Fong H.H.S., Zhang H. (2013). Compounds from the Fruits of the Popular European Medicinal Plant *Vitex Agnus-Castus* in Chemoprevention via NADP(H): Quinone Oxidoreductase Type 1 Induction. Evid. Based Complement. Altern. Med..

[4] Toplan G.G., Ozkan E.E., Taskın T., Abudayyak M., Mat A., Sarıyar G. (2020). Identification, Antioxidant and Cytotoxic Potentials of Casticin in Vitex Agnus-Castus Fruit from Different Geographical Regions of Turkey. Trop. J. Pharm. Res..

[5] Hoberg E., Meier B., Sticher O. (2001). Quantitative High Performance Liquid Chromatographic Analysis of Casticin in the Fruits of Vitex Agnus-Castus. Pharm. Biol..

[6] El-Nawasany L.I. (2019). The Use of Vitex Agnus-Castus to Produce Functional Stirred Yoghurt. J. Food Dairy. Sci..

[7] Ryan S., Ussher J.M., Hawkey A. (2021). Managing the Premenstrual Body: A Body Mapping Study of Women’s Negotiation of Premenstrual Food Cravings and Exercise. J. Eat. Disord..

[8] Gorczyca A.M., Sjaarda L.A., Mitchell E.M., Perkins N.J., Schliep K.C., Wactawski-Wende J., Mumford S.L. (2016). Changes in Macronutrient, Micronutrient, and Food Group Intakes throughout the Menstrual Cycle in Healthy, Premenopausal Women. Eur. J. Nutr..

[9] Zellner D.A., Garriga-Trillo A., Centeno S., Wadsworth E. (2004). Chocolate Craving and the Menstrual Cycle. Appetite.

[10] Rozin P., Levine E., Stoess C. (1991). Chocolate Craving and Liking. Appetite.

[11] Tomelleri R., Grunewald K. (1987). Menstrual Cycle and Food Cravings in Young College Women. J. Am. Diet. Assoc..

[12] Cohen I.T., Sherwin B.B., Fleming A.S. (1987). Food Cravings, Mood, and the Menstrual Cycle. Horm. Behav..

[13] Michener W., Rozin P., Freeman E., Gale L. (1999). The Role of Low Progesterone and Tension as Triggers of Perimenstrual Chocolate and Sweets Craving. Physiol. Behav..

[14] Osman J.L., Sobal J. (2006). Chocolate Cravings in American and Spanish Individuals: Biological and Cultural Influences. Appetite.

[15] di Tomaso E., Beltramo M., Piomelli D. (1996). Brain Cannabinoids in Chocolate. Nature.

[16] Barrientos M.A.E., Maeda J.M.K., Chaves I.E., Tulini F.L., Souza V.B., Thomazini M., da Costa Rodrigues C.E., Favaro-Trindade C.S. (2021). Production of Vitex (*Vitex Agnus-Castus* L.) Extract in Powder Form Using Spray-drying: Potential for the Production of Functional Foods. J. Food Process Preserv..

[17] Fukahori M., Kobayashi S., Naraki Y., Sasaki T., Oka H., Seki M., Masada-Atsumi S., Hakamatsuka T., Goda Y. (2014). Quality Evaluation of Medicinal Products and Health Foods Containing Chaste Berry (*Vitex Agnus-Castus*) in Japanese, European and American Markets. Chem. Pharm. Bull..

[18] Salvim M.O., Thomazini M., Pelaquim F.P., Urbano A., Moraes I.C.F., Favaro-Trindade C.S. (2015). Production and Structural Characterization of Solid Lipid Microparticles Loaded with Soybean Protein Hydrolysate. Food Res. Int..

[19] Xiao Z., Li W., Zhu G. (2015). Effect of Wall Materials and Core Oil on the Formation and Properties of Styralyl Acetate Microcapsules Prepared by Complex Coacervation. Colloid Polym. Sci..

[20] Singleton V.L., Orthofer R., Lamuela-Raventós R.M. (1999). Analysis of Total Phenols and Other Oxidation Substrates and Antioxidants by Means of Folin-Ciocalteu Reagent. Methods Enzymol..

[21] Adamson G.E., Lazarus S.A., Mitchell A.E., Prior R.L., Cao G., Jacobs P.H., Kremers B.G., Hammerstone J.F., Rucker R.B., Ritter K.A. (1999). HPLC Method for the Quantification of Procyanidins in Cocoa and Chocolate Samples and Correlation to Total Antioxidant Capacity. J. Agric. Food Chem..

[22] Alañón M.E., Castle S.M., Siswanto P.J., Cifuentes-Gómez T., Spencer J.P.E. (2016). Assessment of Flavanol Stereoisomers and Caffeine and Theobromine Content in Commercial Chocolates. Food Chem..

[23] Meilgaard M., Civille G.V., Carr B.T. (1999). Sensory Evaluation Techniques.

[24] Dutcosky S.D. (2013). Análise Sensorial de Alimentos.

[25] Mazzocato M.C., Thomazini M., Favaro-Trindade C.S. (2019). Improving Stability of Vitamin B12 (Cyanocobalamin) Using Microencapsulation by Spray Chilling Technique. Food Res. Int..

[26] Tulini F.L., Souza V.B., Echalar-Barrientos M.A., Thomazini M., Pallone E.M.J.A., Favaro-Trindade C.S. (2016). Development of Solid Lipid Microparticles Loaded with a Proanthocyanidin-Rich Cinnamon Extract (*Cinnamomum zeylanicum*): Potential for Increasing Antioxidant Content in Functional Foods for Diabetic Population. Food Res. Int..

[27] Albertini B., Passerini N., Pattarino F., Rodriguez L. (2008). New Spray Congealing Atomizer for the Microencapsulation of Highly Concentrated Solid and Liquid Substances. Eur. J. Pharm. Biopharm..

[28] Zuidam N.J., Shimoni E. (2010). Overview of Microencapsulates for Use in Food Products or Processes and Methods to Make Them. Encapsulation Technologies for Active Food Ingredients and Food Processing.

[29] Gamboa O.D., Gonçalves L.G., Grosso C.F. (2011). Microencapsulation of Tocopherols in Lipid Matrix by Spray Chilling Method. Procedia Food Sci..

[30] Paucar O.C., Tulini F.L., Thomazini M., Balieiro J.C.C., Pallone E.M.J.A., Favaro-Trindade C.S. (2016). Production by Spray Chilling and Characterization of Solid Lipid Microparticles Loaded with Vitamin D3. Food Bioprod. Process..

[31] deMan J.M. (1992). X-ray Diffraction Spectroscopy in the Study of Fat Polymorphism. Food Res. Int..

[32] Tulini F.L., Souza V.B., Thomazini M., Silva M.P., Massarioli A.P., Alencar S.M., Pallone E.M.J.A., Genovese M.I., Favaro-Trindade C.S. (2017). Evaluation of the Release Profile, Stability and Antioxidant Activity of a Proanthocyanidin-Rich Cinnamon (*Cinnamomum zeylanicum*) Extract Co-Encapsulated with α-Tocopherol by Spray Chilling. Food Res. Int..

[33] Chambi H.N.M., Alvim I.D., Barrera-Arellano D., Grosso C.R.F. (2008). Solid Lipid Microparticles Containing Water-Soluble Compounds of Different Molecular Mass: Production, Characterisation and Release Profiles. Food Res. Int..

[34] Okuro P.K., Matos Junior F.E., Fávaro-Trindade C.S. (2013). Technological Challenges for Spray Chilling Encapsulation of Functional Food Ingredients. Food Technol. Biotechnol..

[35] Sillick M., Gregson C.M. (2012). Spray Chill Encapsulation of Flavors within Anhydrous Erythritol Crystals. LWT Food Sci. Technol..

[36] Bianchi O., Dal Castel C., Oliveira R.V.B.d., Bertuoli P.T., Hillig E. (2010). Avaliação Da Degradação Não-Isotérmica de Madeira Através de Termogravimetria-TGA. Polímeros.

